# Impaired glomerular filtration rate, high grade albuminuria and associated factors among adult patients admitted to tertiary Hospital in Ethiopia

**DOI:** 10.1186/s12882-018-1153-5

**Published:** 2018-12-04

**Authors:** Tamiru Adugna, Hailu Merga, Esayas Kebede Gudina

**Affiliations:** 1 Department of Internal medicine, College of Health Sciences, Ambo University, Ambo, Ethiopia; 2 Department of Epidemiology, Institute of Health, Jimma University, Jimma, Ethiopia; 3 Department of Internal medicine, Institute of Health, Jimma University, Jimma, Ethiopia

**Keywords:** Albuminuria, Chronic kidney disease, Creatinine, Glomerular filtration rate, Jimma University medical center

## Abstract

**Background:**

Chronic Kidney Disease (CKD), the worldwide Public Health problem, is also one of the rising non-communicable diseases in low and middle-income countries. Its early detection and treatment using readily available, inexpensive therapies can slow or prevent progression to end-stage renal disease. Hence, this study was aimed at assessing impaired estimated glomerular filtration rate (eGFR), high grade albuminuria, and associated factors among adult patients admitted to Jimma University Medical Center in South west Ethiopia.

**Methods:**

Hospital based cross sectional study was conducted from November 1, 2016 to April 30, 2017. Consecutive sampling method was used to select study participants. Logistic regression analysis was conducted to generate factors associated with impaired estimated GFR and albuminuria. A *P*-value of < 0.05 was considered statistically significant.

**Results:**

The study involved 422 patients admitted to Jimma University Medical Center who had at least one test result for urinalysis and serum creatinine level during the study period. Fifty two (12.3%) of the study subjects had high grade albuminuria, 19.2, 19.4, and 32.7% had impaired estimated glomerular filtration rate according to Modification of Diet in Renal Disease (MDRD-4), Chronic Kidney Disease Epidemiology (CKD-EPI), and Cockcroft-Gault **(**CG) equations respectively. Old age (AOR = 2.4;95%CI:1.4–4.01), male sex (AOR = 2.1;95%CI:1.16–3.7), and hypertension (AOR = 2.23; 95%CI:1.24–4.01) were independently associated with impaired eGFR using one of the two equations while diabetes mellitus (AOR = 2.8; 95%CI:1.33–5.82) and BP measurement above optimal (AOR = 4.7; 95%CI:1.9–11.53) were associated with high grade albuminuria.

**Conclusions:**

High grade albuminuria and impaired eGFR were found in significant proportion of adults admitted to the hospital for various medical conditions. Old age, hypertension, diabetes mellitus and male gender were independently associated with these alterations. These findings necessitate routine urinalysis and estimation of GFR for all hospitalized adults with known CKD risk factors.

## Background

Chronic Kidney Disease (CKD), a worldwide Public Health problem, is a gradual loss in kidney function. It is characterized by reduction in Glomerular Filtration Rate (GFR), increased albumin excretion, or both [1]. It is associated with adverse outcome of kidney failure, cardiovascular disease and premature death [2–6]. The global increase of traditional risk factors are the driven factors for the global increase in magnitude of the CKD [7].

Glomerular Filtration Rate (GFR) is a measure of how well kidneys are cleaning blood-taking out extra water and waste. Specifically, it estimates how much blood passes through the glomeruli each minute [8]. It is central to diagnosis and management of Chronic Kidney Disease and accepted as the best overall measure of kidney function. As the clearance of endogenous toxins is affected by various factors, estimated Glomerular Filtration Rate (eGFR) calculated based on serum creatinine level adjusted for patient’s age, gender and race is often used as surrogate measures of Glomerular Filtration Rate (eGFR) [9].

Besides eGFR, albuminuria which is the abnormal level of protein in the urine has an important prognostic value and should be included in the evaluation of patients with CKD [10, 11]. Albuminuria is the marker of renal injury which has link with kidney disease progression, increased atherosclerosis, and left ventricular abnormalities indirectly contributing to cardiovascular diseases/disorders and death [4–6].

The preventive strategies of CKD involve identifying those at risk of developing CKD; awareness creation for the peoples on how to prevent renal disease; raising the awareness of the general public, policy makers, and health care workers; modifying the lifestyle of susceptible individuals; detecting early stage of CKD; and hindering the progress of the disease [12].

Even though there are evidences suggesting high burden of CKD in Africa [13], there is paucity of data in sub-Saharan Africa in general and Ethiopia in particular. Moreover, the existing data are hampered by poor quality that limits inferences. This calls for more information and validated measures of kidney function especially in the context of the growing burden of non-communicable diseases in the continent [12].

In Ethiopia, the burden of end-stage renal disease is widely felt by both the public and healthcare providers. However, study about CKD is almost non-existent and the real burden of the disease remains unknown. A small scale facility based cross sectional study conducted in southcentral Ethiopia revealed that at least 18% of patients with diabetes on follow up have CKD; most of them have never been diagnosed [14].

Early detection and treatment of CKD slow or prevent progression to end stage renal failure in selected groups of patients. Interventions targeting CKD, particularly to reduce urine protein excretion (ACEI and/or ARB) significantly reduced ESRD risk in patients with High Grade proteinuria [2, 3, 15, 16]. As routine serum creatinine test and urine dipstick are readily available in most public hospitals in Ethiopia, they can be used along with clinical data to screen high risk groups for CKD. The aim of this study was thus to determine the magnitude of impaired glomerular filtration rate, high grade albuminuria, and associated factors among adult patients admitted to Jimma University Medical Center in southwest Ethiopia. Additionally, the concordance among different serum creatinine based GFR estimating equations was assessed.

## Methods

### Study setting

The study was conducted at Jimma University Medical Center (JUMC) between November 1, 2016 and April 30, 2017. JUMC is one of the oldest public hospitals in the Ethiopia located 352 km southwest of Addis Ababa. Currently, it is the only teaching and referral hospital in the southwestern part of the country, providing services for about 15 million peoples. Approximately 17,000 inpatients, 218,000 outpatient visits, 14,000 emergency cases and 6000 deliveries in a year coming to the hospital.

### Study design and data collection

Institutional cross-sectional study was conducted to recruit adult patients admitted to Jimma University Medical Center during the study period. However, only adults with at least one test result for Renal Function Test (RFT) and urinalysis were included in this study. Patients with possibility of functional proteinuria, and those with established pre-eclampsia/eclampsia were excluded from study.

The sample size was calculated using a formula for estimation of single population proportion taking prevalence of impaired eGFR and albuminuria to be 50%, margin of error of 5%, and using 95% confidence level. Finally, considering the expected patient loss from the study as 10%, the final sample size was calculated to be 422. Consecutive sampling method was used to recruit those study participants.

Data were collected from patients and their record using structured questionnaire which was subdivided into four parts; socio-demographic characteristics, medical history, physical examination and laboratory findings, was developed in English with modification from Screening and Early Evaluation of Kidney Disease (SEEK) study [17]. All laboratory tests were done following the standard procedures recommended by the manufacturer. Serum creatinine was determined using fully automated machine (Horiba Ltd., Japan) and ABX Pentra Creatinine 120 CP reagent using Jaffe’s reaction by laboratory technicians. Quality control test was done for all tests and results were used for analysis only for those who passed the test. Moreover, all laboratory tests at the hospital are validated continuously by external quality assurance done by Ethiopian Public Health Institute. Data was collected by trained nurses and medical interns with supervision by internal medicine resident from Jimma University Medical Center. During preparatory stage, the questionnaires was carefully designed and pre-tested on 5% of study population to minimize errors. Based on the result of pretest, revision was made on few contents and concepts of the questionnaire. Data collectors and supervisors were trained for two days prior to data collection period. The collected data was checked for consistency and completeness immediately at the end of the interview.

### Measurements

Acute kidney injury was defined in the study as an increase in serum creatinine (SCr) by > = 0.3 mg/dl within 48 h; and/or an increase in SCr to > = 1.5 times baseline, which is known or presumed to have occurred within the prior 7 days; and/or urine volume < 0.5 ml/kg/h for 6 h. Chronic kidney disease was defined as abnormal serum creatinine, eGFR and/or proteinuria which persisted for > 3 months or eGFR < 60 ml/min in patients with ultrasonographic finding of shrunken kidney and/or normocytic normochromic anemia. On the other hand, alcohol use problem was defined as drinking alcohol with ‘yes’ response at least to one of the CAGE assessment question. Smoking status was measured as a person who had used at least 100 cigarettes during their lifetime and who at the time they participated in the study, reported smoking every day or some of the day. Elevated serum creatinine was measured as serum creatinine level > 1.01 mg/dl for female and > 1.25 mg/dl for male using NHANES-III cut off point for blacks and end stage renal disease/chronic kidney failure as eGFR< 15 ml/min using steady state serum creatinine [18]. Impaired estimated glomerular filtration rate (eGFR) was measured in our study as eGFR less than 60 ml/min using one of the three creatinine based equations; Cockcroft-Gault (CG), Modification of Diet in Renal Disease (MDRD-4) or Chronic Kidney Disease Epidemiology (CKD-EPI). High grade albuminuria was also measured as dipstick proteinuria > = 1+ excluding functional proteinuria. Khat chewing (*Catha edulis)* an evergreen plant that is extensively cultivated in the highlands of Ethiopia and surrounding countries, was also measured. A patient was considered a chewer if s/he respond ‘yes’ to the question ‘Did you ever chew khat?”

### Data processing and analysis

Collected data was checked for completeness by principal investigator. Serum creatinine based glomerular filtration rate was estimated for all study participants by CG, MDRD-4 and CKD-EPI equations using QxMD calculator with correction for black race. Urinalysis of all study subjects were revised; after excluding possible causes of functional albuminuria, dipstick proteinuria of > = + 1 was taken as albuminuria. Finally, the data was entered in to the computer using EpiData software and after verification; it was exported to SPSS (IBM SPSS Statistics for Macintosh, Version 20.0. Armonk, NY) for analysis. Descriptive statistics like percentages, means, medians, standard deviations and ranges were used to describe findings.

A bi-variate analysis was done to sort variables candidate for multiple logistic regression having value less than or equals to 0.25. Multiple logistic regression analyses were conducted using Backward LR to generate factors associated with the dependent variable. *P*-value < 0.05 and 95% confidence interval (CI) and AOR was used in judging the statistical significance of the associations between independent variables and the outcome variable.

## Results

### Socio-demographic characteristics of study participants

A total of 422 patients were included in the study. About half of the participants (50.9%) were male. The mean age of the study participants was 45.37 (SD = 18.49); 29.4% were 60 years and older. One hundred ninety one (45.3%) couldn’t read and write while those who had completed college and above were less than 10 %. About 60% of them were rural residents and 38.4% of them were farmers (Table 1).

**Table 1:** Socio-demographic characteristics of study participants, Jimma University Medical Center, Ethiopia, 2017

Socio-demographic characteristics	Category	Frequency	Percentage
Age	<40 year	175	41.5
	40–59 year	122	28.9
	> = 60 year	125	29.6
	Mean ± SD	45.37 ± 18.5	
Sex	Male	215	50.9
	Female	207	49.1
Marital status	Married	343	81.3
	Single	54	12.8
	Widowed	16	3.8
	Divorced	9	2.1
Religion	Muslim	293	69.4
	Christian	129	30.6
Occupation	Farmer	162	38.4
	Housewife	105	24.9
	Merchant	63	14.9
	Student	40	9.5
	Employee	27	6.4
	Daily laborer	12	2.8
	Others	13	3.1
Educational status	Illiterate	191	45.3
	Grade 1–8	167	39.6
	Grade 9–12	31	7.3
	College/University	33	7.8
Residence	Rural	254	60.2
	Urban	168	39.8

### Clinical and lifestyle characteristics of study participants

Majority of patients (~ 85%) have only one urine analysis which is both microscopic and dipstick examination of random urine sample. For those having urine analysis more than once, we have used the last one in our study. Majority (89.8%) of the study participants were medical patients. Of this 71.3% had non-communicable disease (NCD) as one of their major diagnosis including 12.8% patients who were diagnosed with AKI and/or CKD during their hospital stay. The rest of patients (10.2%) were surgical patients including 10 (2.4%) who were post-operation state; however, all laboratory data was determined before surgery.

**Fig. 1:**
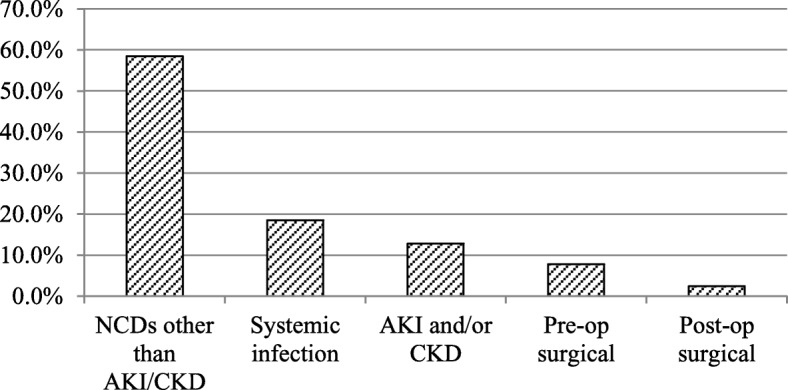
Admission diagnosis of study participants, Jimma University Medical Center, Ethiopia, 2017

Forty one percent of study participants had BP measurement above optimal at admission; of which 27% were previously diagnosed to have hypertension. Forty nine (11.6%) were not checked for hypertension previously of which 4 (8.2%) were diagnosed to have hypertension during admission. Fifty four (12%) were known diabetic patients. One hundred forty nine (35.3%) study participants had used Khat; of which about 15.9% were using it at the time of this study. About one third (33.4%) of study participants were engaged in regular physical exercise or their work involve significant physical activities. The study revealed that about 6 % (5.9%) of patients were obese and about 6 % (6.2%) of study subjects were HIV patients (Table 2 and Fig. 1).

**Table 2:** Life style and clinical characteristics of study participants, Jimma University Medical Center, Ethiopia, 2017

Life style and clinical characteristics	Category	Frequency	Percentage
Cigarette smoking	Never	400	94.8
	Past	17	4.0
	Current	5	1.2
Alcohol drinking	Never	378	89.6
	Occasionally	25	5.9
	< 3 times/week	13	3.1
	3–6 times/week	4	0.9
	Daily	2	0.5
Alcohol use problem	No	413	97.9
	Yes	9	2.1
Khat chewing	Never	273	64.7
	Past	82	19.4
	Current	67	15.9
NSAIDs use with two weeks	No	354	83.9
	Yes	68	16.1
History of DM	No	368	87.2
	Yes	54	12.8
History of hypertension	No	259	61.4
	Yes	114	27.0
	Not checked for previously	49	11.6
Physical exercise	No	204	48.3
	< 150 min/week	77	18.2
	> = 150 min/week	141	33.4
BP	< 120/80	249	59.0
	120–139/80–89	57	13.5
	> = 140/90	116	27.5
BMI	< 25	397	94.1
	> = 25	25	5.9
HIV status	Nonreactive	396	93.8
	Reactive	26	6.2

### Pattern of **impaired eGFR and albuminuria**

One hundred seventeen (27.7%) of the study subjects had elevated serum creatinine level; 138 (32.7%), 81 (19.2%) and 82(19.4% had impaired estimated GFR by CG, MDRD-4 and CKD-EPI equations respectively. The study revealed that about 12 % (12.3%) of the study participants had albuminuria. Moreover, 21.7 and 32.1% of the study participants with impaired eGFR using CG and MDRD-4 equation respectively had albuminuria (Table 3).

**Table 3:** Pattern of high grade albuminuria according to level of eGFR, Jimma University Medical Center, Ethiopia, 2017

	eGFR by CG equation		eGFR by MDRD-4 equation	
	< 60 ml/min (*n* = 138)	> = 60 ml/min (*n* = 284)	< 60 ml/min/1.73 m2 (*n* = 81)	> = 60 ml/min/1.73 m2 (*n* = 341)
Albuminuria (> = + 1)	30 (21.7%)	22 (7.7%)	26 (32.1%)	26 (7.6%)

### Factors associated with impaired eGFR and albuminuria

Logistic regression analysis was conducted to see the association between dependent and independent variables. Accordingly, on bivariate analysis to look for candidate variables for final model; age (COR = 3.6; 95%CI: 2.15–5.9), sex (COR = 2.1; 95%CI: 1.4–3.16), residence (COR = 2.01;95%CI:1.30–3.1), educational status (COR = 0.46; 95% CI: 0.31–0.7), occupational status (COR = 0.55:95%CI:0.36–0.83), Khat chewing (COR = 1.7;95% CI:1.11–2.56), hypertension (COR = 2.3;95%CI:1.53–3.5), and physical exercise (COR = 1.6;95%CI:1.02–2.54) were associated with impaired eGFR using CG equations (Table 4).

**Table 4:** Bi-variate logistic regression analysis of factors associated with impaired eGFR using CG-equation, Jimma University Medical Center, Ethiopia, 2017

Variables		Impaired eGFR by CG			
		Yes n = 138	No n = 284	COR (95%CI)	*P*-value
Age	> = 60 year	60 (43.5%)	65 (22.9%)	3.56 (2.146, 5.920)	.000
	40-59 year	42 (30.4%)	80 (28.2%)	2.027 (1.201, 3.421)	.008
	<40 year	36 (26.1%)	139 (48.9%)	1	
Sex	Male	87 (63.0%)	128 (45.1%)	2.079 (1.370, 3.156)	.001
	Female	51 (37.0%)	156 (54.9%)	1	
Residence	Rural	98 (71.0%)	156 (54.9%)	2.01 (1.3, 3.108)	.002
	Urban	40 (29.0%)	128 (45.1%)	1	
Formal education	Yes	58 (42%)	173 (60.9%)	0.465 (.308, .703)	.000
	No	80 (58%)	111 (39.1%)	1	
Occupation	Non-farmer	72 (52.2%)	189 (66.5%)	0.548 (.362, .830)	.005
	Farmer	66 (47.8%)	95 (33.5%)	1	
Cigarette smoking	Yes	11 (8.0%)	11 (3.9%)	2.150 (.908, 5.089)	.082
	No	127 (92.0%)	273 (96.1%)	1	
Khat chewing	Yes	60 (43.5%)	89 (31.3%)	1.685 (1.108, 2.564)	.015
	No	78 (56.5%)	195 (68.7%)	1	
Physical exercise	> = 150 min/week	55 (39.9%)	86 (30.3%)	1.610 (1.021, 2.538)	.040
	< 150 min/week	25 (18.1%)	52 (18.3%)	1.210 (.687, 2.131)	.509
	No	58 (42.0%)	146 (51.4%)	1	
History of known HTN	Yes	72 (52.2%)	91 (32.0%)	2.31 (1.525, 3.510)	.000
	No	66 (47.8%)	193 (68.0%)	1	
BP (mmHg)	> = 140/90	54 (39.1%)	62 (21.8%)	2.805 (1.758, 4.476)	.000
	120–139/80–89	25 (18.1%)	32 (11.3%)	2.52 (1.382, 4.581)	.003
	< 120/80	59 (42.8%)	190 (66.9%)	1	

After multivariable logistic regression analysis using Backward LR, old age (AOR = 2.376, 95%CI 1.38–4.1), male gender (AOR = 1.61, 95% CI:1.03–2.5), rural residence (AOR = 1.9, 95%CI:1.18–3.0), and elevated BP (hypertension) (AOR = 1.97, 95%CI:1.14–3.4) were independently associated with impaired eGFR computed using CG equation (Table 5). On the other hand, age (COR = 1.87;95%CI: 1.04–3.36), sex (COR = 2.1;95%CI:1.26–3.5), residence (COR = 2.53;95%CI: 1.45–4.42), occupation (COR = 0.564;95%CI: 0.35–0.92) and hypertension (COR = 3.6;95%CI: 2.1–6.1) were associated with impaired eGFR computed by MDRD-4 equation on bi-variate analysis (Table 6). After multivariable logistic regression analysis; male gender (AOR = 2.1, 95%CI: 1.2–3.7), rural residence (AOR = 2.95;95%CI: 1.6–5.6), doing moderate exercise (AOR = 2.3; 95%CI: 1.12–4.7), and hypertension (AOR = 2.6; 95%CI:1.4–4.9) were associated independently with impaired eGFR using MDRD-4 equation (Table 7).

**Table 5:** Multi-variable logistic regression analysis of factors associated with impaired eGFR using CG equation, Jimma University Medical Center, Ethiopia, 2017

		COR (95%CI)	AOR (95%CI)	*P*-value
Age	> = 60 year	3.564 (2.146, 5.920)	2.376 (1.378, 4.095)	0.002
	40-59 year	2.027 (1.201, 3.421)		
	<40 year	1		
Sex	Male	2.079 (1.370, 3.156)	1.609 (1.029, 2.515)	0.037
	Female	1		
Residence	Rural	2.01 (1.3, 3.108)	1.882 (1.181, 3.000)	0.008
	Urban	1		
BP (mmHg)	> = 140/90	2.805 (1.758, 4.476)	1.974 (1.142, 3.411)	0.015
	120–139/80–89	2.516 (1.382, 4.581)	2.112 (1.114, 4.025)	0.022
	< 120/80	1

**Table 6:** Bi-variate logistic regression analysis of factors associated with impaired eGFR by MDRD-4 equation, Jimma University Medical Center, Ethiopia, 2017

Variables		Impaired eGFR by MDRD			
		Yes n = 81	No n = 341	COR (95% CI)	*P*-value
Age	> = 60 year	25 (30.9%)	100 (29.3%)	1.433 (.783, 2.623)	0.244
	40–59 year	30 (37.0%)	92 (27.0%)	1.869 (1.040, 3.358)	0.036
	< 40 year	26 (32.1%)	149 (43.7%)	1	
Sex	Male	53 (65.4%)	162 (47.5%)	2.091 (1.262, 3.465)	0.004
	Female	28 (34.6%)	179 (52.5%)	1	
Residence	Rural	62 (76.5%)	192 (56.3%)	2.532 (1.451, 4.419)	0.001
	Urban	19 (23.5%)	149 (43.7%)	1	
Formal education	Yes	38 (46.9%)	193 (56.6%)	.678 (.417, 1.102)	0.117
	No	43 (53.1%)	148 (43.4%)	1	
Occupational status	Farmer	40 (49.4%)	121 (35.5%)	1	
	Others	41 (50.6%)	220 (64.5%)	.564 (.346, .919)	0.022
Cigarette smoking	Yes	7 (.6%)	15 (4.4%)	2.056 (.810, 5.221)	0.130
	No	74 (91.4%)	326 (95.6%)	1	
Physical exercise	> = 150 min	31 (38.3%)	110 (32.3%)	1.515 (.875, 2.622)	0.138
	< 150 min/week	18 (22.2%)	59 (17.3%)	1.640 (.857, 3.137)	0.135
	No	32 (39.5%)	172 (50.4%)	1	
History of known HTN	Yes	43 (53.1%)	113 (33.1%)	3.254 (1.971, 5.374)	0.000
	No	38 (47.9%)	228 (66.9%)	1	
NSAIDs use within two weeks	Yes	8 (9.9%)	60 (17.6%)	.513 (.235, 1.121)	0.094
	No	73 (90.1%)	281 (82.4%)	1	
BP (mmHg)	> = 140/90	39 (48.1%)	77 (22.6%)	3.562 (2.079, 6.103)	0.000
	120–139/80–89	11 (13.6%)	46 (13.5%)	1.682 (.788, 3.588)	0.179
	< 120/80	31 (38.3%)	218 (63.9%)	1

**Table 7:** Multi-variable logistic regression analysis of factors associated with impaired eGFR by MDRD-4 equation, Jimma University Medical center, Ethiopia, 2017

Variables				*P*-value
		COR (95% CI)	AOR (95% CI)	
Sex	Male	2.091 (1.262, 3.465)	2.084 (1.167, 3.721)	0.013
	Female	1	1	
Residence	Rural	2.532 (1.451, 4.419)	2.954 (1.556, 5.609)	0.001
	Urban	1		
Physical exercise	> = 150 min	1.515 (.875, 2.622)	.902 (.473, 1.721)	0.754
	< 150 min/week	1.640 (.857, 3.137)	2.290 (1.120, 4.685)	0.023
	No	1		
History of known HTN	Yes	3.254 (1.971, 5.374)	2.233 (1.244, 4.010)	0.007
	No	1		
BP (mmHg)	> = 140/90	3.562 (2.079, 6.103)	2.597 (1.378, 4.893)	0.003
	120–139/80–89	1.682 (.788, 3.588)	1.676 (0.757, 3.706)	0.203
	< 120/80	1		

On bi-variate analysis being obesity (COR = 3.04; 95%CI: 1.2–7.7), having history of known hypertension (COR = 3.2; 95%CI: 1.7–5.9), and diabetes mellitus (COR = 3.1; 95%CI: 1.5–6.1) were associated with albuminuria (Table 8). After multi-variate logistic regression analysis, we found that diabetes mellitus (AOR = 2.8, 95%CI:1.3–5.8) and hypertension (AOR = 6.303, 95%CI: 3.1–12.99) were strongly associated with high grade albuminuria (Table 9).

**Table 8:** Bi-variate logistic regression analysis of factors associated with high grade albuminuria, JUMC, Ethiopia, 2017

Variables		High grade albuminuria			
		Yes *n* = 52	No *n* = 370	COR (95% CI)	*P*-value
Age	> = 60 year	14 (26.9%)	111 (30.0%)	.977 (.473, 2.019)	0.951
	40–59 year	18 (34.6%)	104 (28.1%)	1.341 (.677, 2.657)	0.400
	< 40 year	20 (38.5%)	155 (41.9%)	1	
Sex	Male	32 (61.5%)	183 (49.5%)	1.635 (.902, 2.96)	0.105
	Female	20 (38.5%)	187 (50.5%)	1	
Alcohol drinking	Yes	9 (17.3%)	35 (9.5%)	2.0 (.902, 4.451)	0.088
	No	43 (82.7%)	335 (90.5%)	1	
BMI	> = 25	7 (13.5%)	18 (4.9%)	3.04 (1.204, 7.68)	0.019
	< 25	45 (86.5%)	352 (95.1%)	1	
History of known HTN	Yes	33 (63.5%)	130 (35.1%)	3.206 (1.754, 5.86)	0.000
	No	19 (36.5%)	240 (64.9%)	1	
History of DM	Yes	14 (26.9%)	40 (10.8%)	3.039 (1.517, 6.09)	0.002
	No	38 (73.1%)	330 (89.2%)	1	
BP (mmHg)	> = 140/90	29 (55.7%)	87 (23.5%)	6.583 (3.21, 13.47)	0.000
	120–139/80–89	11 (21.2%)	46 (12.4%)	4.723 (1.96, 11.3)	0.001
	< 120/80	12(23.1%)	237(64.1%)	1

**Table 9:** Multi-variable logistic analysis of factors associated with high grade albuminuria, Jimma University Medical Center, Ethiopia, 2017

Variables		OR with 95% CI		
		COR (95% CI)	AOR (95%CI)	*P*-value
History of DM	Yes	3.039 (1.517, 6.091)	2.785 (1.332, 5.825)	0.006
	No	1		
BP (mmHg)	> = 140/90	6.583 (3.21, 13.474)	6.303 (3.059, 12.987)	0.000
	120–139/80–89	4.723 (1.965, 11.352)	4.757 (1.962, 11.533)	0.001
	< 120/80	1		

### Performance of serum creatinine based equations

**Fig. 2:**
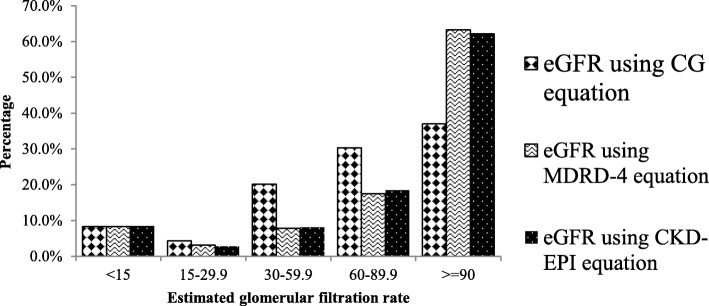
Estimated GFR using serum creatinine based equations, Jimma University Medical Center, Ethiopia, 2017

**Fig. 3:**
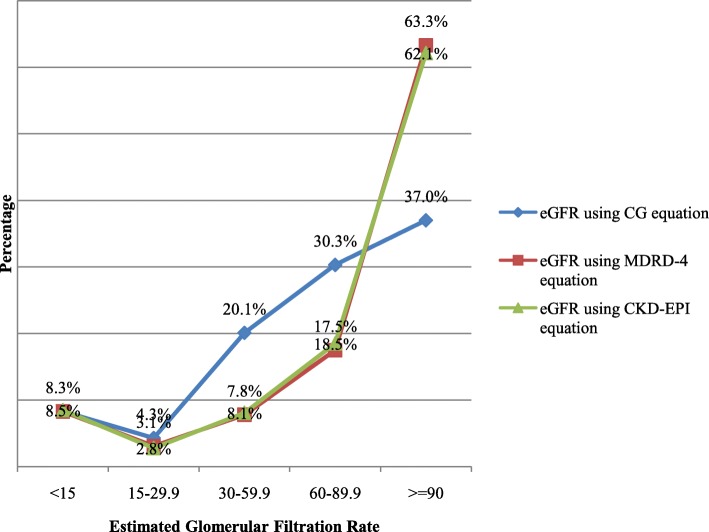
Comparison of the three serum creatinine based equations, Jimma University Medical Center, Ethiopia, 2017

The study showed that the mean SCr was 1.83 mg/dl whereas the mean of eGFR by CG, MDRD-4 and CKD-EPI of study subjects were 78.47, 109.4 and 97.34 respectively. The three serum creatinine based equations performance correlate better for low eGFR (Figs. 2-3).

## Discussion

This study revealed that about 28 % (27.7%) of the patients had elevated serum creatinine level above the cut-off point for blacks. The prevalence of impaired estimated glomerular filtration rate (eGFR < 60 ml/min) was 19.2, 19.4 and 32.7% by MDRD-4, CKD-EPI and CG equations respectively. This result may not reflect the real burden of CKD both in the study setting as well as in the country as it likely included non-steady serum creatinine. A similar study conducted among diabetic patients attending hospital at Southern part of Ethiopia found eGFR < 60 ml/min/1.73 m2 of 18.2 and 23.8% according to the MDRD and Cockcroft-Gault (CG) equations [14].

The other systematic and meta-analysis in which hospital based studies were excluded, estimates the prevalence of CKD in Sub Saharan Africa ranged from 2% in Cote d’Ivoire to 30% in Zimbabwe with overall prevalence of 13.9% which is not in line with our finding [12]. The observed difference might be due to differences in study design, population and study setting. The magnitude of impaired eGFR found in this study is comparable with that found by SEEK study [17].

Prevalence of dipstick albuminuria found by our study (12.3%) roughly goes in line with the study done in tertiary hospital in Lagos, Nigeria which found dipstick proteinuria in 8.3% of HIV sero-negative and 42.5% of HIV positive subjects [18].

Old age (*P* = .002, AOR = 2.376), and hypertension (*P* = .015, AOR = 1.974) were independently associated with two fold increased risk of having impaired eGFR by CG equation. This is consistent with other similar studies [19, 20].

Our study has also shown that rural residence (*P* = 0.001, AOR = 2.954) and male gender (*P* = 0.013, AOR = 2.084) were associated with lower eGFR by both CG and MDRD-4 equations. Most studies show that the risk of CKD increases in urban settings than rural residents [21]. The risk with rural residence in our study may be due to poor living standard and increased risk of infection both of which beget higher risk of glomerulonephritis. However, there are no good quality data that compare differences with residence in Africa [12]. Similarly, the link with male sex is contrary to previous reports which have shown higher eGFR impairment in female [14, 17, 19].

Moderate physical exercise (*P* = .023, AOR = 2.29) was associated with 2 folds increased risk of having impaired eGFR computed by MDRD-4 equation. This finding is inconsistent with studies from North India and Screening and Early Evaluation of Kidney Disease (SEEK) study [17]. This might be due to selection bias.

Individuals who had diabetes mellitus (*P* = .006, AOR = 2.785) were about three times more likely to have high grade albuminuria. On the other hand, BP measurement above optimal (*P* = .001, AOR = 4.757) had increased risk of high grade albuminuria by more than four folds [2, 4, 19, 22, 23].

However, cigarette smoking (*P* = .082), alcohol (*P* = .088), NSAIDs (*P* = .094) and HIV/AIDS (*P* = .605) were not associated with any of the study outcomes likely due to small sample size.

In this study, the prevalence of impaired eGFR using CG-equation is higher than that of impaired eGFR by MDRD-4 and CKD-EPI equations. On the other hand, MDRD-4 and CKD-EPI equations performed closely. This is roughly in line with the study from South-Africa which found that highest agreement between GFR estimators was between MDRD and CKD-EPI equations [12, 17, 22].

More than 70% of the study participants had Non-Communicable Diseases (NCDs) as one of their admission diagnoses. Forty one percent (41%) of patients had BP above optimal (BP > =120/80 mmHg) of which more than 27% were hypertensive while at least 12% of the study participants had diabetes mellitus. On the other hand, we found that more than 19% of the study participants had eGFR < 60 ml/min by all three equations while more than 12% had dipstick proteinuria. These are the reflection of double burden of NCDs on developing nations like Ethiopia where there are limited facilities to care for chronic diseases like ESRD. These all necessitates timely detection and treatment of NCDs in general and CKD in particular and their risk factors.

Even though this study has many strengths, it has few limitations. As the study design used was institution based cross sectional study, it is impossible to infer for the general population. The other limitation is that our serum creatinine based eGFR calculation likely included unsteady serum creatine level. There is also a possibility of functional proteinuria like orthostatic proteinuria as we used random urine sample. Notwithstanding these limitations, we believe that our study has very important findings in the study area and areas with similar set up.

## Conclusions

Impaired estimated Glomerular Filtration Rate and High grade albuminuria of all grades was found in significant proportion of study participants which necessitates routine urine analysis and estimation of Glomerular Filtration Rate for patients with CKD risk factors. On the other hand, MDRD-4 and CKD-EPI equations perform comparable in estimating GFR through the range of eGFR while CG equation correlate better with other equations when eGFR below 30 ml/min. Our study showed that old age, being male sex, and hypertension were independently associated with impaired eGFR using one of the two equations while being diabetic and having BP measurement above optimal were associated with high grade albuminuria. Therefore, Health workers should be vigilant in utilizing available resources to detect risk factors of NCDs including CKD and foster healthy life style of their clients through health education. Estimation of GFR and urine analysis should be routine for patients with traditional risk factors for CKD and urologic diseases.

## Abbreviations

ACR: Albumin-creatinine ratio
AKI: Acute Kidney Injury
BMI: Body Mass Index
CAGE: Cut down, Annoyed, Guilty, Eye opener
CG: Cockcroft-Gault equation
CKD: Chronic kidney disease
CrCl: Creatinine clearance
CVD: Cardiovascular disease
DKA: Diabetic Keto-acidosis
DM: Diabetic mellitus
eGFR: Estimated glomerular filtration rate
ESRD: End-stage renal disease
FBS: Fasting blood sugar
GFR: Glomerular filtration rate
JUMC: Jimma University Medical Center
KDOQI: Kidney Disease Outcomes Quality Initiative
MDRD: Modified Diet Renal Disease
RFT: Renal function test
SCr: Serum creatinine
WHO: World Health Organization
